# Terahertz Irradiation Promotes Angiogenesis in vitro by Enhancing Permeability of the Voltage-Gated Calcium Channel

**DOI:** 10.1371/journal.pone.0317426

**Published:** 2025-02-21

**Authors:** Jichang Li, Hongjie Guo, Linxia Tan, Meng Chen, Xin Wang, Yucun Liu, Shanwen Chen, Yingxin Wang, Hao Yu, Pengyuan Wang

**Affiliations:** 1 Department of Gastrointestinal Surgery, Peking University First Hospital, Beijing, China; 2 Division of Vascular Interventional, Peking University First Hospital, Beijing, China; 3 National Engineering Research Center for Dangerous Articles and Explosives Detection Technologies, Department of Engineering Physics, Tsinghua University, Beijing, China; 4 Weimai Qingtong Medical Technology Co., Wuxi, China; Parul University Parul Institute of Technology, INDIA

## Abstract

Terahertz (THz) waves, positioned between microwave and infrared in the electromagnetic spectrum, have promising applications in medical imaging and biomedicine. In this study, terahertz irradiation at 2.52 THz (100 mW/cm^2^) did not alter the proliferation of human umbilical vein endothelial cells (HUVECs), but significantly enhanced their angiogenic capacity. This enhancement was accompanied by increased levels of angiogenesis-related proteins such as VEGF in the culture supernatant. ATAC sequencing and RNA sequencing revealed a significant increase in the expression of cytoskeleton-associated genes, including PDXP and SH3BP1, post-irradiation. Additionally, intracellular calcium concentration, closely linked to angiogenesis, markedly increased following terahertz exposure. However, diltiazem significantly mitigated the enhanced angiogenic capacity induced by terahertz irradiation. In conclusion, terahertz irradiation promotes angiogenesis in HUVECs, partly by activating the VEGF signaling pathway through increased calcium fluxes.

## Introduction

Terahertz is an electromagnetic wave with a spectrum between microwave and infrared, and its frequency range is 0.1–10 THz (3.3–330 cm^-1^)[1]. Terahertz technology has not been fully developed or applied due to the lack of suitable light sources and detectors, leading scientists to refer to it as the "Terahertz Gap". However, recent advances in laser and semiconductor technologies have spurred significant progress in this area. In particular, graphene-based nano-rectifiers have garnered increasing attention for their potential in terahertz signal rectification and detection. Several studies have demonstrated that devices such as graphene-based three-terminal junctions (G-TTJ) and Y-junctions (G-YJ) can perform nonlinear rectification, frequency multiplication, and terahertz energy harvesting at a frequency of 0.637 THz [2, 3]. These developments have opened new avenues for terahertz communication and signal processing[4, 5], and terahertz technologies are now being progressively applied in areas such as military, security detection, and communications [6–9]. Terahertz can penetrate substances or materials that are opaque to visible and infrared light, such as plastics, ceramics, and insulating foams. As a result, terahertz imaging technology is well-suited for security inspection through packaging materials. This offers several unique advantages for applications in non-invasive national security, packaged goods inspection, and remote environmental sensing[10–12]. The frequency of terahertz waves is 1–4 orders of magnitude higher than that of microwaves, offering a significantly higher information capacity. As a result, terahertz radiation has been developed as a potential frequency band for wireless communications[13].

Due to its unique sensitivity to molecular dynamics, non-ionizing low photon energy, and strong interaction with water, terahertz radiation holds significant promise in medical imaging. Several studies have demonstrated that terahertz imaging can more effectively delineate tumorous and normal tissues in various types of cancer, including colorectal, breast, and skin tumors[14–16]. Terahertz has also been utilized to determine the extent of the burn wound[17]. In addition, the internal atomic vibrations of amino acid molecules, intermolecular hydrogen bonds, van der Waals forces, and low-frequency vibrations of the crystal lattice all fall within the terahertz frequency range[18, 19]. Therefore, terahertz irradiation may induce changes in molecular structural properties, potentially affecting biological functions. It has been reported that the resonance frequency of chemical bonds between the methyl group and DNA in cancer cells lies within the range of approximately 1.6–1.7 THz. Terahertz irradiation has been shown to reduce the degree of methylation in melanoma cells[20]. Previous studies have suggested that terahertz irradiation can regulate gene expression, stimulate specific signaling pathways, and alter cell membrane permeability[21–24]. The experiments *in vivo* also showed that pulsed terahertz wave (2.7 THz, 4 μs pulsewidth, 61.4 μJ per pulse, 3 Hz repetition) irradiating the mouse’s ear skin induced acute inflammatory response[25]. Previous studies have shown that terahertz irradiation has different biological effects on different cells (Table 1)[24, 26–30]. However, the effects of terahertz irradiation on angiogenesis and the underlying molecular mechanisms remain unclear.

In this study, we applied 2.52 THz (100 mW/cm^2^) irradiation to HUVECs to investigate the effects of terahertz radiation on angiogenesis and explore its underlying mechanisms.

**Table 1:** Effects of terahertz radiation on different cells.

Terahertz Sources	Irradiation Parameters	Experimental Object	Biological Effects
Gas laser	3.68 THz,20 mW, 30 mim	erythrocyte	Reduced stability of cell membranes to hypotonic stress
Nonlinear differential frequency mechanism	1.7 THz, 2.4 mW/cm2, 30 min	Blood cancer cells	The degree of DNA methylation is reduced
Gas laser	2.52 THz, 636 mW/cm2, 30~50 min	Jurkat cells	Activates plasma membrane genes and intracellular signal transduction pathways
Free electron laser	0.1~0.15 THz, 0.4 mW/cm2, 20 min	Fibroblasts	Actin polymerization is enhanced
Echo oscillator	0.16THz,50 mW; 0.17THz,10 mW, 6、60 min	Rat neuron-like cells	Neurotransmitter content decreases, protein expression levels change
Optically pumped gas body terahertz laser	2.52 THz,3 mW/cm2, 2、9 h	Mouse mesenchymal stem cells	The expression of some genes is up-regulated or down-regulated without obvious temperature changes

## Materials and Methods

### Cell lines and reagents

The human umbilical vein endothelial cell line (HUVEC) was purchased from the National Biomedical Laboratory Cell Resource Bank. HUVECs were cultured in complete modified DMEM medium (Thermo Fisher Scientific) supplemented with 10% fetal bovine serum (FBS, Thermo Fisher Scientific) and 1% penicillin/streptomycin (PS, Thermo Fisher Scientific). The cells were maintained at a 37°C and 5% CO_2_ atmosphere.

### Cell counting kit-8 assay

HUVEC cells were seeded into 96-well plates (10^4^ cells/well). Cell viability was measured using Cell Counting Kit (CCK)-8 assay kits (LABLEAD, cat # PA137267). The assay was performed by adding CCK8 reagent at a 10% ratio to HUVEC cells culture medium. The absorbance was recorded at a wavelength of 490 nm after incubation for 60 min at 37°C.

### Tube formation assay

The tube formation assay was performed according to the method described by DeCicco-Skinner et al[31]. Briefly, a mixture containing 25 ul of Matrigel and 25 ul of medium is added to the wells of the 96-well plate, and the plate is incubated at 37°C for 1 h. Next, HUVEC cells are placed in the 96-well plate (5×10^4^/well) and after 6 h incubation in a 37°C cell culture incubator, images were captured using an inverted microscope (Olympus, Japan). The number of junctions, meshes, and segment length of the tube were measured by software ImageJ (National Institutes of Health, USA) with the “Angiogenesis Analyzer” plugin.

### Real-time quantitative polymerase chain reaction (RT-qPCR)

Total RNA of cells was extracted using TRIzol one-step method (TRIzol reagent; Invitrogen, USA) according to the manufacturer’s instructions. The Complementary DNA (cDNA) was transcribed from RNA using PrimeScript RT Reagent Kit (Vazyme, China). PowerUp SYBR Green Master Mix (Vazyme, China) was determined the relative expression levels of VEGF. Data were analyzed using the 2^-△△Ct^ method, with GADPH serving as an internal control. Primers sequence were as follows:

**Table:** 

Human GAPDH-F:	GGAGCGAGATCCCTCCAAAAT
Human GAPDH-R:	GGCTGTTGTCAACTTCTCATGG
Human VEGF-F:	GGGCAGAATCATCACGAAGTG
Human VEGF-R:	CACCAGGGTCTCGATTGGAT

### Enzyme-linked immunosorbent assay

The VEGF levels in cell supernatants were detected by ELISA kits (MULTISCIENCES, Zhejiang, China) according to the manufacturer’s protocols. Briefly, add 100μl of the supernatant to each well of the plate, followed by the addition of 50 μl of detection antibody working solution per well. Incubate on a shaker at 200 rpm at room temperature for 2 hours. Discard the liquid and wash each well with 300 μl of wash buffer, repeating the wash step 6 times. Subsequently, add 100μl of streptavidin working solution to each well and incubate on a shaker at 200 rpm at room temperature for 45 minutes. Discard the liquid and wash each well with 300μl of wash buffer, repeating the wash step 6 times. Add 100 μl of the substrate solution to each well and incubate in the dark at room temperature (25°C ± 3°C) for 5 to 30 minutes. And then, add 100μl of stop solution to each well. Finally, the plates were read at a wavelength of 450 nm using a microplate reader (Bio-Rad).

### Western Blot

HUVEC cells were lysed using RIPA buffer with protease inhibitors, and protein concentrations were quantified by BCA assay. Equal amounts of protein (30–50 µg) were separated by SDS-PAGE and transferred to PVDF membranes. Membranes were blocked with 5% non-fat milk in TBST, followed by overnight incubation with primary antibodies against (anti-VEGF, ABclonal). After washing, membranes were incubated with HRP-conjugated secondary antibodies and detected using an ECL detection kit. Protein bands were visualized using a chemiluminescence system.

### Human Angiogenesis Proteome Profiler

The Proteome Profiler Human Angiogenesis Array Kit (R&D Systems, Minneapolis, MN, USA) was used detected the relative levels of selected angiogenesis-related proteins in the cell supernatants according to the manufacturer’s protocols. Briefly, the supernatant of cells was mixed with the biotinylated detection antibody and incubated with the array membrane at 4°C overnight. Then, after 30 min incubation of HRP-conjugated secondary antibodies, the captured proteins were visualized with chemiluminescence solution. Then, the intensity of spots was analyzed using ImageJ (version 2.3.0).

### Face scan Confocal Ca^2+^ Imaging of Cytosolic Ca^2+^

After terahertz irradiation, HUVEC cells were loaded with the calcium indicator flou-4 AM (5uM, Thermo Fisher Scientific) for 30 min and then Two-dimensional (2D) confocal Ca^2+^-imaging was performed. Ca^2+^ changes were recorded in HUVECs loaded with Fluo-4 AM. For Fluo-4, excitation and emission wavelengths were 488 nm and >515 nm, respectively. An Olympus confocal microscope (Olympus Corp, Tokyo, Japan, with FluoView 1000) equipped with a ×20 objective lens and an argon-ion laser were used. Additionally, we used a high-content imaging system (MD IXM-4) to observe real-time changes in intracellular calcium ions and analyzed the data with ImageJ.

### Nuclei Isolation and ATAC-seq

Cells were harvested from cell culture and lysed in lysis buffer. The Nextera DNA Library Preparation Kit (Illumina) was used to perform the transposition according to the manufacturer’s manual. 50000 nuclei were pelleted and resuspended with transposase, for 30 minutes at 37°C. The transposed DNA fragments were purified immediately after with a MinElute PCR Purification Kit (Qiagen). After Samples were PCR-amplified using 1X NEBNext High-Fidelity PCR Master Mix (New England Biolabs, MA). Subsequent libraries were purified with the MinElute PCR Purification Kit (Qiagen). Sequencing was performed using the Illumina NovaSeq 6000 platform with a PE150 strategy. Raw reads in fastq format were processed to remove contaminating adapter sequences using Trimmomatics. Quality distribution plot and base content distribution were generated by FASTQC. ATAC-seq reads were aligned to the hg38 genome using BWA (0.7.13-r1126). Only uniquely mapping reads with at most two mismatches were retained. Peaks were called uing MACS v2.1.2 with the significance cut-off q-value < = 0.05. bigWig files were generated using the deeptools. Peak files were generated using MACS v2 with default settings. The samples had 67 to 90 million pass filter reads with more than 95% of bases above the quality score of Q30. The ATAC-seq data for the HUVEC cell lines were deposited in the NCBI Gene Expression Omnibus (GEO) database under accession number GSE250026.

### RNA sequencing and Analysis

Total RNA was isolated and purified from the samples using TRIzol (Thermo Fisher, 15596018) following the manufacturer’s instructions. Quality control for RNA quantity and purity was performed using the NanoDrop ND-1000 (NanoDrop, Wilmington, DE, USA), and RNA integrity was assessed with the Bioanalyzer 2100 (Agilent, CA, USA). Samples with concentrations >50 ng/μL, RIN values >7.0, and total RNA >1 μg were deemed suitable for downstream experiments. Polyadenylated mRNA was specifically captured using oligo(dT) magnetic beads (Dynabeads Oligo (dT), cat.25-61005, Thermo Fisher, USA) through two rounds of purification. Finally, paired-end sequencing (PE150) was conducted on the Illumina NovaSeq 6000 platform (LC Bio Technology CO., Ltd., Hangzhou, China) following standard procedures. The samples had 39 to 50 million pass filter reads with more than 95% of bases above the quality score of Q30. The RNA sequencing (RNA-seq) data for the human breast cancer cell lines were deposited in the NCBI’s GEO database under accession number GSE248763. Differential expression analysis was done using DESeq2 package in R. Differential expression genes (DEG) were filtered by a *P*value < 0.05, the absolute value of logFC > = 0.585. The ClusterProfiler package was used for enrichment analysis.

### Flow Cytometric Detection of Reactive Oxygen Species (ROS)

Cells were harvested and washed with phosphate-buffered saline (PBS) to remove any residual medium. The ROS-sensitive fluorescent dye, dichlorofluorescein diacetate (DCFH-DA), was added to the cell suspension at a final concentration according to the manufacturer’s instructions. Cells were incubated in the dark at 37°C for 30 minutes to allow for dye uptake and ROS detection. After incubation, cells were washed with PBS to remove any unincorporated dye, and then resuspended in PBS for flow cytometric analysis.

### Data availability

The RNA-seq data generated from this study have been uploaded to GEO with the accession number GSE248763. The ATAC-seq data generated from this study have been uploaded to GEO with the accession number GSE250026.

### Statistical analysis

GraphPad 9.0 software was used for statistical analysis. The Mann–Whitney test and one-way ANOVA were performed to analyze data from two groups and more than two groups, respectively. Results were presented as the means ± SD. P< 0.05 were considered statistically significant.

## Results

### Experimental setup and thermal considerations

**Fig 1:**
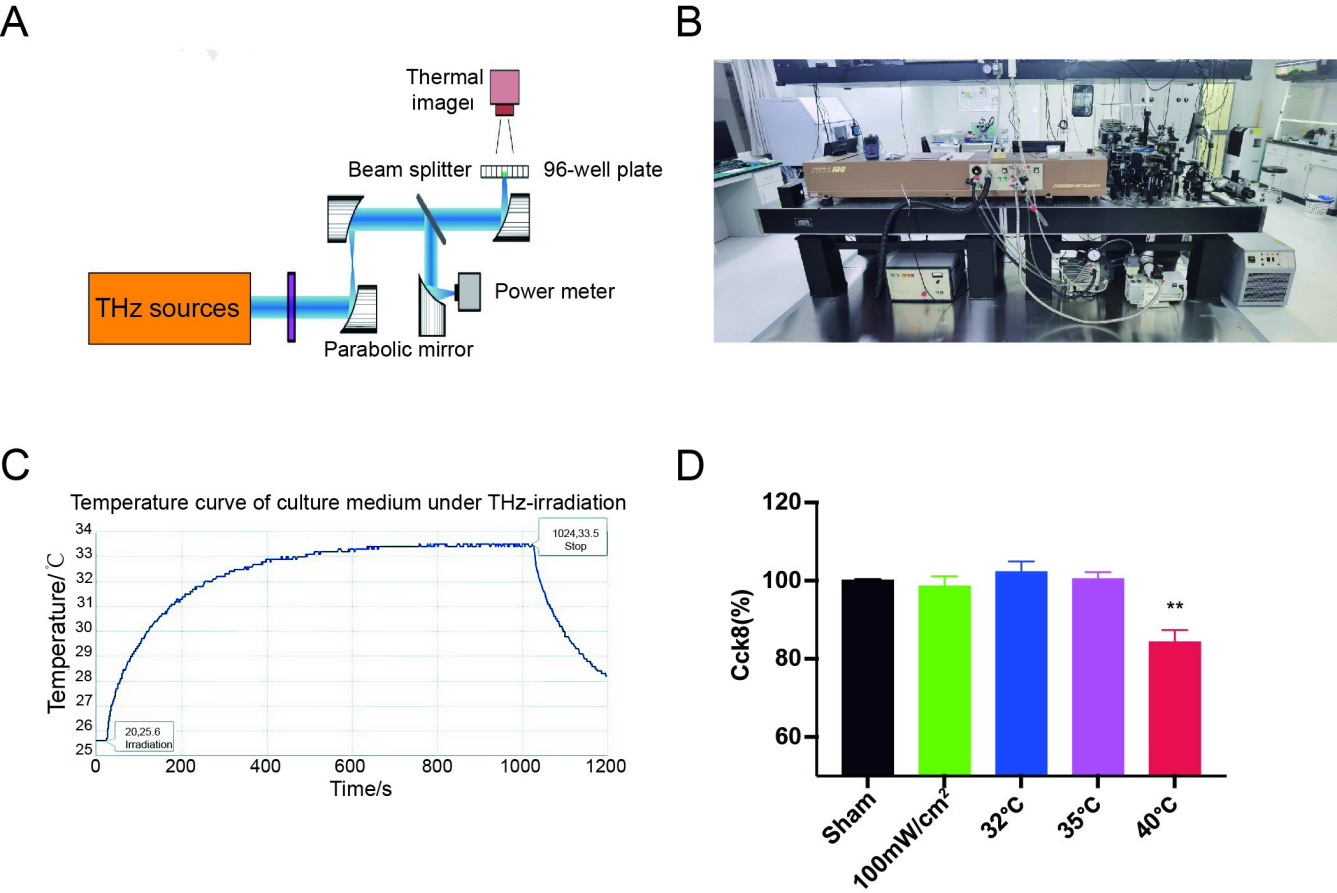
Experimental setup and thermal considerations. (A) Schematic representation of our custom terahertz exposure system. (B) Reality images of our custom THz exposure system. (C) Temperature curve of culture supernatant under terahertz irradiation. (D) CCK8 assay detected the activity of cells after heat treatment at different temperatures.

We seeded human umbilical vein endothelial cells (HUVECs) in 96-well plates containing culture medium and exposed them to 2.52 THz radiation at a power density of 100 mW/cm² (Fig 1A and 1B). To minimize thermal stress from the slight temperature increase during THz exposure, the room temperature was maintained at 26.5 ± 0.5°C. The temperature of the culture medium was monitored throughout irradiation, and the temperature change curve is shown in Fig 1C. During terahertz exposure, the medium temperature gradually increased, ultimately stabilizing at 33°C. Control groups consisted of identical cell plates under the same conditions but without irradiation. To observe the thermal effects on cells, we subjected the cells to heat treatment alone and found that at temperatures of 32°C and 35°C, cell viability did not change significantly. However, when the medium temperature reached 40°C, cell viability decreased significantly (Fig 1D). These results suggest that the thermal effect of the terahertz irradiation conditions used in this study had minimal impact on cell viability.

### Terahertz irradiation had no effect on the proliferation of HUVECs

**Fig 2:**
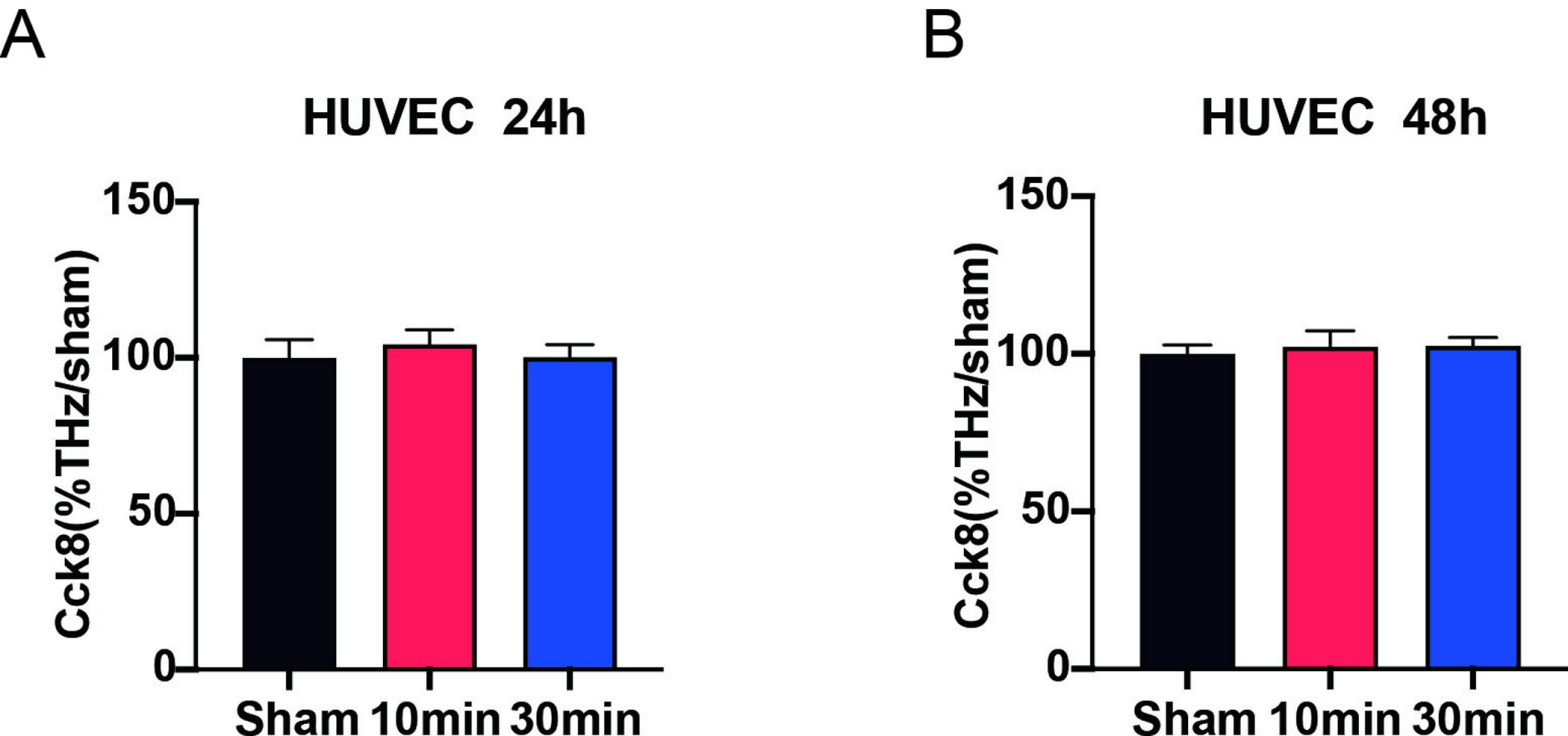
Terahertz irradiation had no effect on the proliferation of HUVECs. (A-B) HUVECs were irradiated for 10 min or 30 min with terahertz (2.52 THz,100 mW/cm2), and detected the proliferation of cells after incubation at 37°C for 24h and 48 h, respectively.

Proliferation is one of the important physiological functions of living cells, and is the basis for the growth, development, reproduction and heredity of organisms. In order to detect whether terahertz irradiation affects the proliferation of HUVECs, referring to previous studies[32], we irradiated HUVECs cells with THz for 10 min or 30 min, and detected the proliferation of cells after incubation at 37°C for 24h and 48h, respectively. We found that terahertz irradiation had no significant effect on the proliferation of HUVECs (Fig 2).

### Terahertz irradiation promotes the angiogenic effect of HUVECs

**Fig 3:**
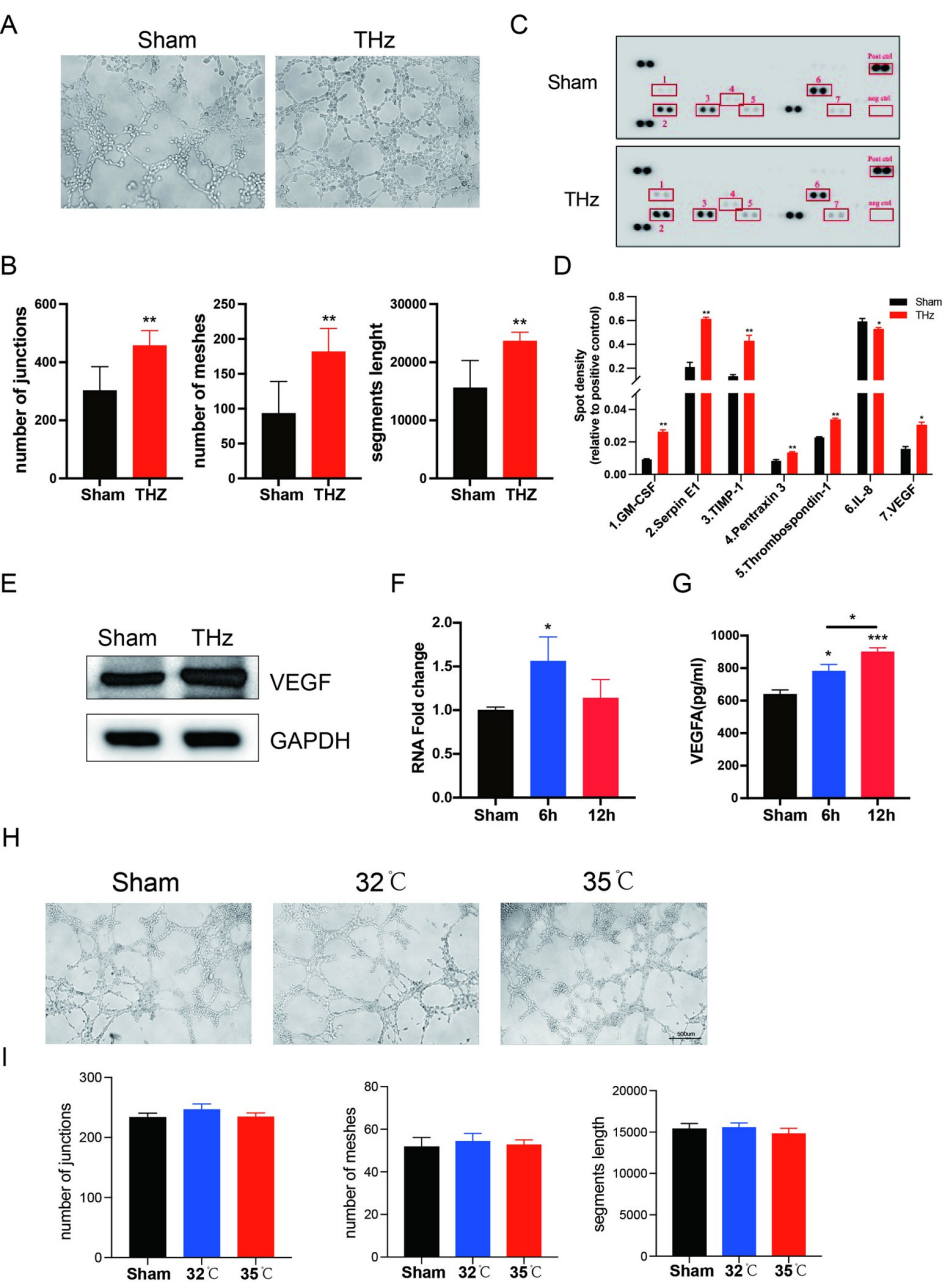
Terahertz irradiation promotes the angiogenic effect of HUVECs. (A) Representative images of tube formation assay of HUVECs after terahertz irradiation. (B) Average numbers of junctions and meshes and the segments length formed by HUVECs in different groups. (C) Representative images of the Proteome Profiler Human Angiogenesis Array. Spots with significant differences are marked with red boxes. Pos ctrl indicates the positive control, and neg ctrl indicates the negative control. (D) The densities of spots with significant differences. (E) Western blot (WB) was performed to detect the protein levels of VEGF in HUVEC cells after terahertz irradiation. (F) mRNA expression levels of VEGF in HUVECs after terahertz irradiation. (G) the level of VEGF in culture supernatant after terahertz irradiation. (H) Representative images of tube formation assay of HUVECs after heating. (I) Average numbers of junctions and meshes and the segments length formed by HUVECs in different groups. * P < 0.05 vs. the control group, ** P < 0.01 vs. the Sham group.

Angiogenesis plays a critical role in metabolism and development, with endothelial cells serving as the fundamental building blocks of blood vessels. Tube formation is a key biological function of HUVECs. Our results demonstrate that, compared to the control group, terahertz irradiation significantly enhanced the angiogenic capacity of HUVECs (Fig 3A), as evidenced by an increased number of junctions and segment lengths (Fig 3B). However, lower-power terahertz irradiation did not promote angiogenesis (S1C and S1D Fig), suggesting that the irradiation energy of terahertz is an important factor influencing its biological effects. Consistent with these findings, proteome array analysis revealed that several angiogenesis-related cytokines, including VEGF, were upregulated at 6 hours after terahertz irradiation (Fig 3C and 3D). Western blot analysis also confirmed an increase in VEGF levels following irradiation (Fig 3E). Both VEGF mRNA levels and VEGF content in the supernatant were increased at 6 hours post-irradiation. However, at 12 hours post-irradiation, the VEGF mRNA levels gradually returned to baseline, while the VEGF content in the supernatant remained elevated. This suggests that the changes in VEGF levels within the cells and in the supernatant are not entirely consistent. (Fig 3F and 3G). To investigate whether the thermal effects of terahertz irradiation promote angiogenesis, we subjected HUVECs to heat treatment alone. The results showed that heating alone did not enhance angiogenic capacity (Fig 3H and 3I). Furthermore, terahertz irradiation did not significantly affect intracellular reactive oxygen species (ROS) levels, indicating that it did not induce a cellular stress response (S1A and S1B Fig). Collectively, these findings suggest that terahertz irradiation enhances tubule formation in HUVECs by increasing VEGF levels.

### Terahertz irradiation increases intracellular calcium ion concentration

**Fig 4:**
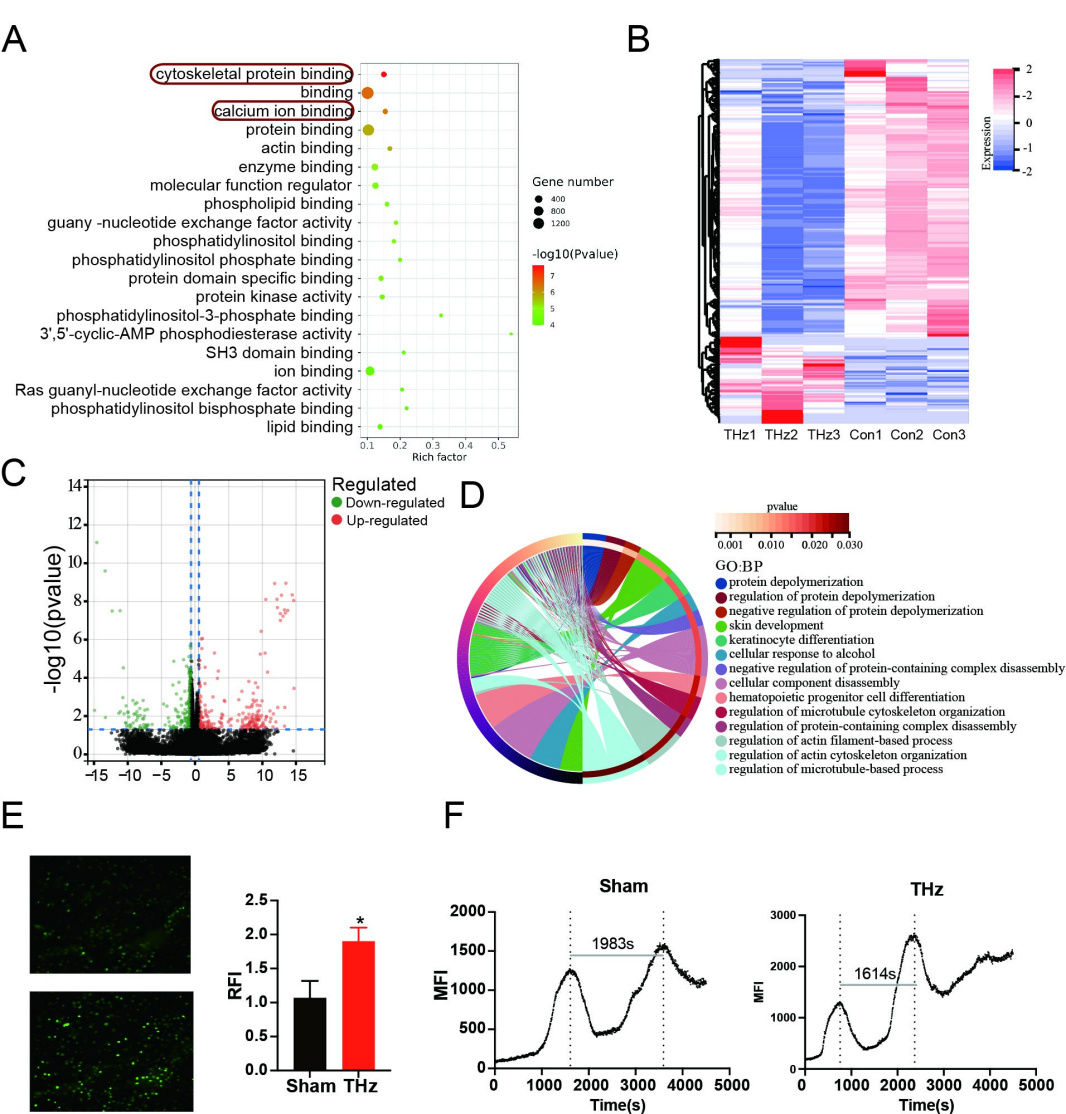
Terahertz irradiation increases intracellular calcium ion concentration. (A) GO enrichment results of ATAC sequencing. (B)Differentially expressed genes in Sham group and THz group. (C) Volcano plots showing the results of differentially expressed genes. (D) GO enrichment results of RNA sequencing (E) Fluorescent staining of intracellular calcium ions in different groups and statistical analysis of their relative fluorescence intensity (RFI). (F) Intracellular calcium oscillations in HUVECs after terahertz irradiation.

To further investigate the mechanisms underlying the enhanced tubular formation and VEGF secretion following terahertz irradiation, we assessed chromatin accessibility in HUVECs. ATAC-seq analysis revealed significant increases in chromatin accessibility associated with calcium ion binding and cytoskeletal protein binding after terahertz exposure (Fig 4A). Additionally, transcriptome sequencing identified 1,121 differentially expressed genes, with 865 downregulated and 265 upregulated (Fig 4B and 4C, P-value < 0.05, logFC ≥ 0.585). Gene Ontology (GO) enrichment analysis indicated a strong association with biological processes related to the cytoskeleton (Fig 4D), consistent with the ATAC-seq findings. Calcium ions act as second messengers in various cellular processes and are closely linked to biological functions, including the regulation of angiogenesis and gene expression[33, 34]. Previous reports indicated that terahertz irradiation can enhance the permeability of the voltage-gated calcium channel[35]. ATAC-seq also indicated changes in genes associated with intracellular calcium ion binding. To validate this observation, we performed intracellular calcium staining using Fluo-4 AM. Fluorescent imaging revealed a significant increase in intracellular calcium concentrations following terahertz irradiation (Fig 4E), and the frequency of calcium oscillations was also notably accelerated (Fig 4F). These results suggest that terahertz irradiation enhances the tube-forming ability of HUVECs, likely by elevating intracellular calcium ion concentrations.

### Diltiazem inhibits the proangiogenic effects of terahertz

**Fig 5:**
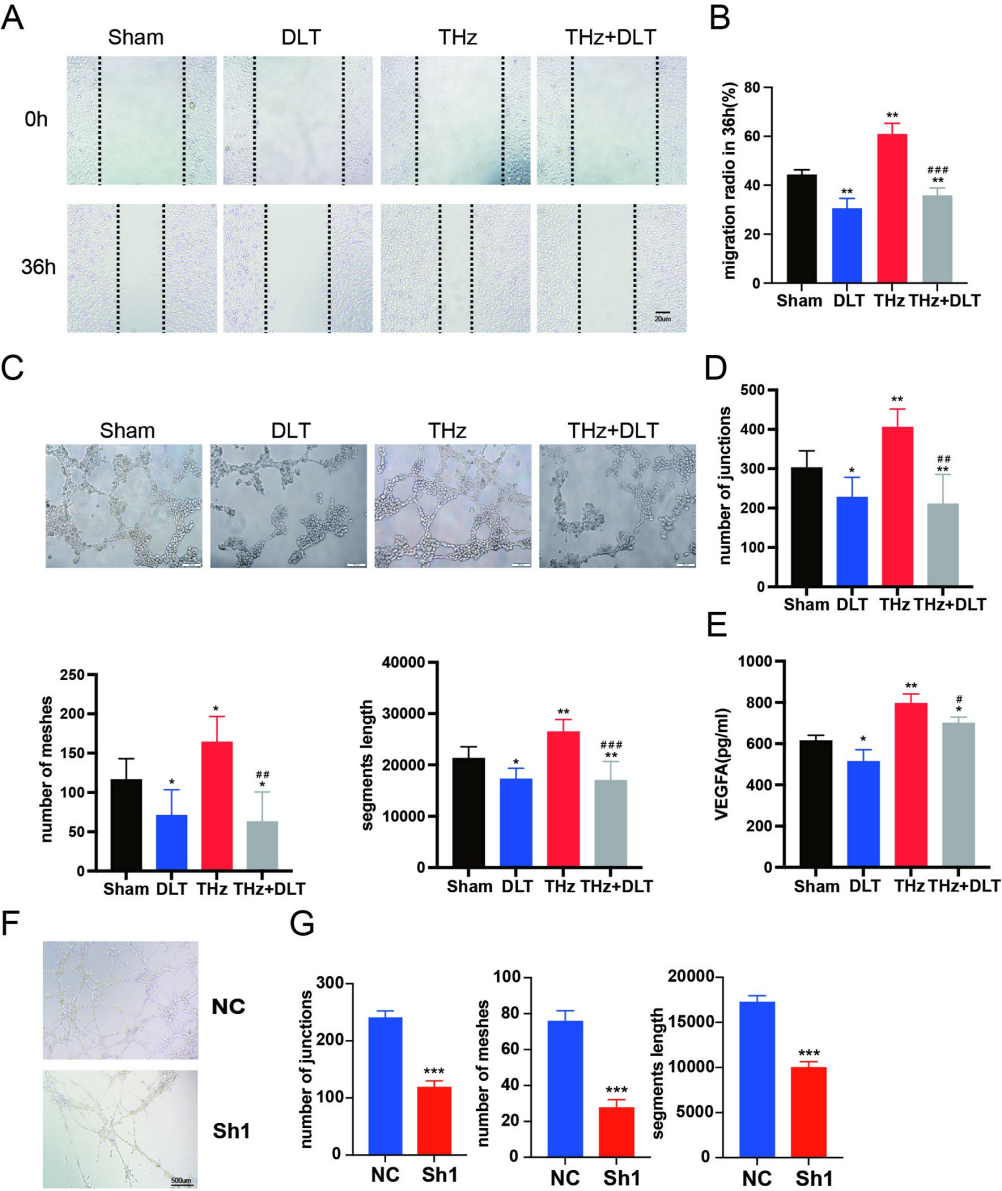
Diltiazem inhibits the proangiogenic effects of THz. (A) Representative images of the scratch in different groups of cells. (B)Migration rate of HUVECs at the edge of scratches in different groups in the scratch assay. (C) Representative images of tube formation assay of HUVECs after THz irradiation. (D) Average numbers of junctions and meshes and the segments length formed by HUVECs in different groups. (E) The level of VEGF in culture supernatant in different groups after THz irradiation. *P < 0.05, **P < 0.01vs. the Sham group. #P < 0.05, ##P < 0.01, ###P < 0.001 vs. the THz group.

To investigate the role of calcium ions in promoting angiogenesis under terahertz exposure, we used diltiazem, an inhibitor of voltage-gated calcium (CaV) channels, to block intracellular calcium influx. The results showed that terahertz irradiation enhanced the migration capacity of HUVECs, and this effect was significantly inhibited by diltiazem (Fig 5A and 5B). Similarly, diltiazem also suppressed the increased tube formation ability induced by terahertz exposure (Fig 5C and 5D). Furthermore, elevated VEGF levels in the cell supernatant after terahertz irradiation were reversed by diltiazem treatment (Fig 5E). To further support these findings, we cultured HUVECs in media containing varying calcium ion concentrations. The results indicated that increasing calcium ion concentration enhanced VEGF secretion, providing additional evidence for the role of calcium ions in VEGF release and angiogenesis (S1E–S1G Fig). Taken together, these findings suggest that terahertz irradiation promotes VEGF release and angiogenesis by increasing intracellular calcium concentrations.

## Discussion

Terahertz imaging has garnered significant attention due to its ability to penetrate nonpolar and non-metallic materials, as well as its label-free, non-invasive, and non-ionizing properties, making it ideal for obtaining interior information from biological samples[36]. Terahertz imaging can act as a sensitive hydration probe in biological tissue and other materials[37]. Recent advances have also highlighted the use of terahertz absorbance for detecting cell viability and chemical-cell interactions, demonstrating the advantages of being both label-free and contact-free[38, 39]. While significant progress has been made in using terahertz for cell imaging and detection, the biological effects of terahertz on various tissues remain poorly understood. Interestingly, recent studies have shown that terahertz exposure can enhance neuronal synaptic transmission and promote oligodendrocyte differentiation, possibly through alterations in neuronal gene expression dynamics[40]. In this study, we investigated the effects of terahertz radiation on the proliferation and angiogenesis capacity of HUVECs in vitro. Our results indicate that 2.52 THz irradiation enhanced the vascularization capacity of HUVECs, with increased calcium flux potentially contributing to this effect. Terahertz exposure also induced changes in the expression of several angiogenesis-related genes, and an increased secretion of VEGF was observed in HUVECs following irradiation. These findings suggest that terahertz irradiation may serve as a non-invasive approach to modulate the activity of vascular endothelial cells. However, further validation in clinically relevant models, both in vitro and in vivo, is needed.

In this study, stable terahertz irradiation was generated using a far-infrared gas laser, and cells cultured in 96-well plates were exposed to the radiation for specified time periods. A thermal imager was used to detect any potential thermal effects of the terahertz irradiation. Additional experiments were conducted by incubating cells at increasing temperatures, and the results showed that the thermal effects from the terahertz irradiation used in this study had minimal impact on the proliferation of HUVECs. Furthermore, 2.52 THz irradiation at 100 mW/cm² did not affect HUVEC proliferation. We then investigated the influence of different terahertz power levels on the angiogenesis capability of HUVECs. The results demonstrated that higher-power irradiation (100 mW/cm^2^) promoted tube formation, characterized by increased junctions and segment length in the HUVECs. No significant side effects were observed at this power level, as intracellular ROS levels did not exhibit notable changes.

Using a Proteome Profiler Human Angiogenesis Array, we demonstrated that multiple angiogenesis-related molecules, including VEGF, were significantly upregulated after terahertz irradiation. We further validated this by showing an increase in VEGF mRNA expression following irradiation. Importantly, the levels of secreted VEGF in the culture supernatant were also elevated after terahertz exposure. These findings suggest that the increased expression and secretion of VEGF may be one of the mechanisms through which terahertz irradiation promotes angiogenesis in HUVECs. Additionally, enhanced calcium fluxes have been identified as the upstream mechanism responsible for VEGF’s effect on vascular endothelial cells[34, 41]. Interestingly, ATAC sequencing revealed that both cytoskeletal protein binding and calcium ion binding signaling pathways were among the most significantly altered pathways following terahertz irradiation. Previous studies have also highlighted the close relationship between calcium ions and angiogenesis[42]. Li, Jun et al. demonstrated that calcium ions play a crucial role in the expression of key angiogenic factors, such as HIF-1α and VEGF, through activation of the CaMKII-CREB pathway[32]. This suggests that the increase in intracellular calcium ion concentration could be a key mechanism by which terahertz irradiation enhances the angiogenic capacity of HUVECs. To test this hypothesis, we employed a calcium ion fluorescent probe, which revealed that terahertz irradiation significantly elevated intracellular calcium levels and altered calcium oscillation patterns. Subsequently, diltiazem, a calcium channel blocker, was used to inhibit the calcium flux induced by terahertz irradiation. The results showed that diltiazem effectively attenuated the enhanced angiogenesis capacity of HUVECs following terahertz exposure. To further explore the role of calcium ion concentration in angiogenesis, we cultured HUVECs in media containing varying concentrations of calcium ions. The results demonstrated that limiting calcium ion availability significantly impaired both the tube formation ability of HUVECs and VEGF secretion. The increased calcium levels induced by terahertz irradiation, and their subsequent effect on VEGF secretion via the CaMKII-CREB or PI-3K/Akt pathways, warrant further investigation.

In conclusion, our findings demonstrate that 2.52 THz terahertz irradiation at 100 mW/cm^2^ promotes angiogenesis in HUVECs in vitro, partly through activation of the VEGF signaling pathway and modulation of calcium fluxes. These results suggest that terahertz irradiation could offer a novel approach for intervening in angiogenesis and warrants further exploration as a potential therapeutic strategy.

## Limitations of this study

This study has several limitations. First, in vivo experiments were not performed to validate the in vitro findings. Future studies will address this gap by evaluating the effects of terahertz irradiation in animal models. Additionally, further investigations are needed to explore the impact of terahertz irradiation across a broader spectrum on angiogenesis.

## Supporting information

S1 Fig Detection of ROS levels and tube formation ability in HUVEC cells. (A and B) Flow cytometry detection of intracellular ROS levels in HUVEC cells after terahertz irradiation. (C) Representative images of tube formation assay of HUVECs after THz irradiation. (D) Average numbers of junctions and meshes and the segments length formed by HUVECs in different groups. (E) Representative images of tube formation assay of HUVECs under different calcium ion concentrations. (F) Average numbers of junctions and meshes and the segments length formed by HUVECs in different calcium ion concentrations. (G) VEGF levels in the supernatant under different calcium ion concentrations.
(TIF)
