# Supplementary figures and images for: Terahertz Irradiation Promotes Angiogenesis in vitro by Enhancing Permeability of the Voltage-Gated Calcium Channel
Source: PLoS One. 2025 Feb 21;20(2):e0317426. doi: 10.1371/journal.pone.0317426 (PMC11844849; doi:10.1371/journal.pone.0317426)

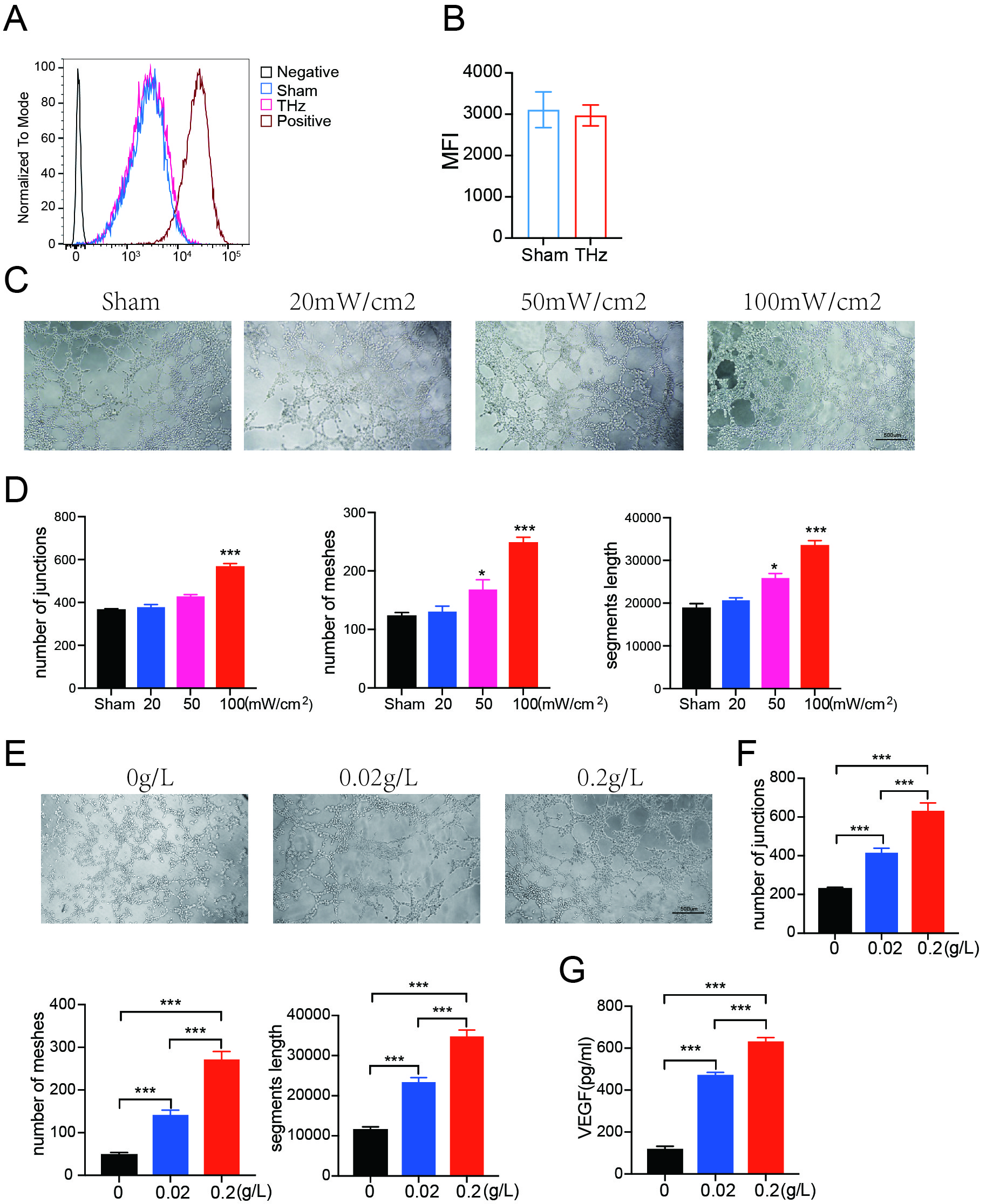

Supplement: S1 Fig — (A and B) Flow cytometry detection of intracellular ROS levels in HUVEC cells after terahertz irradiation. (C) Representative images of tube formation assay of HUVECs after THz irradiation. (D) Average numbers of junctions and meshes and the segments length formed by HUVECs in different groups. (E) Representative images of tube formation assay of HUVECs under different calcium ion concentrations. (F) Average numbers of junctions and meshes and the segments length formed by HUVECs in different calcium ion concentrations. (G) VEGF levels in the supernatant under different calcium ion concentrations. (TIF) [file pone.0317426.s001.tif]
